# Serial interval in households infected with SARS-CoV-2 variant B.1.1.529 (Omicron) is even shorter compared to Delta

**DOI:** 10.1017/S0950268822001248

**Published:** 2022-08-05

**Authors:** Matthias an der Heiden, Udo Buchholz

**Affiliations:** Department for Infectious Disease Epidemiology, Robert Koch-Institute, Berlin, Germany

A previous publication of the authors has shown a significant shortening of the serial interval – the time from symptom onset of the primary case to symptom onset of secondary cases – in household clusters from wild-type to Alpha and Delta variant of concern (VOC) (4.8; 4.5; 4.0 days) [1]. Since December 2021 the VOC Omicron is circulating in Germany, and since calendar week (CW) 2 it surpassed a level of 80% of infections [2]. As Omicron was ‘spreading at a rate … not seen with any previous variant’ [3] it was unclear if the rapidity of the rise in case numbers was due to an increase of transmissibility or a shortening of the serial interval, or both.

To extend the findings from our previous paper also to the period when Omicron dominated we aimed to investigate the timing of symptom onset of secondary household cases also for Omicron households.

We used the same methodology as in our publication [1]. Briefly, we used data from the mandatory reporting system requiring to report laboratory-confirmed cases with SARS-CoV-2 infection to the Robert Koch-Institute (RKI). (Not more than six) cases belonging to the same household and likely having been infected within the household were grouped as household clusters. Household size or composition was not reported. We analysed data of household clusters with information on symptom onset of all cases involved. We included cases that were reported to RKI from the beginning of the pandemic until 24 May 2022. For this analysis we defined first cases as those with the first symptom onset. Other household cases with the same date of symptom onset were excluded from analysis because we could not differentiate if this case was a co-primary case infected externally or a secondary case truly infected within the household. We defined the serial interval of household cases as the interval of symptom onset of the first case to symptom onset of any other reported household case. We included only time periods when the respective variants dominated defined as weeks when the proportion of randomly sequenced strains exceeded 80%. Thus, we attributed infections to the wild-type from CW 10/2020 until 48/2020, to Alpha from CW 12/2021 until 22/2021, to Delta from CW 27/2021 until 49/2021 and to Omicron from CW 02/2022 until 16/2022. To ensure rather complete reporting of the household cases we included only households for which the primary case had a symptom onset at least 28 days before 24 May 2022. To display differences of the serial interval of the variants we estimated the cumulative probability function of the serial interval by variant. To explore the effect of vaccination we also compared the intervals from symptom onset to the ensuing household cases when the first household case was vaccinated or not vaccinated. To compare the difference of the mean interval of secondary cases' symptom onset after symptom onset of primary cases statistically we used the Kruskal–Wallis test. Analysis was performed with Stata (Stata Corporation 2021, Stata Statistical Software: Release 17. College Station, TX, USA: StataCorp LLC).

We included 16 964, 22 185, 39 277 and 11 512 household clusters during dominance of the wild-type, Alpha, Delta and Omicron, respectively. In total, 25 364 (31%) of 81 475 clusters comprised only two cases. First cases were a mean of 42, 39, 34 and 31 years old, and secondary cases a mean of 37, 34, 31 and 31 years during wild-type, Alpha, Delta and Omicron, respectively. Vaccination status of the first case affected the mean of the serial interval by less than 10% and inconsistently.

Figure 1a shows that the distribution of observed serial intervals could be described by a *γ* distribution for all variants. The maximum height of the *γ* distribution increases for wild-type, Alpha and Delta to Omicron from 15% to 20%. The *γ* distribution would also expect a certain number of secondary cases with symptom onset on day 0, but this number is substantially lower than one we observed – in line with our assumption that many of those cases had been exposed outside of the household.

**Fig. 1.**
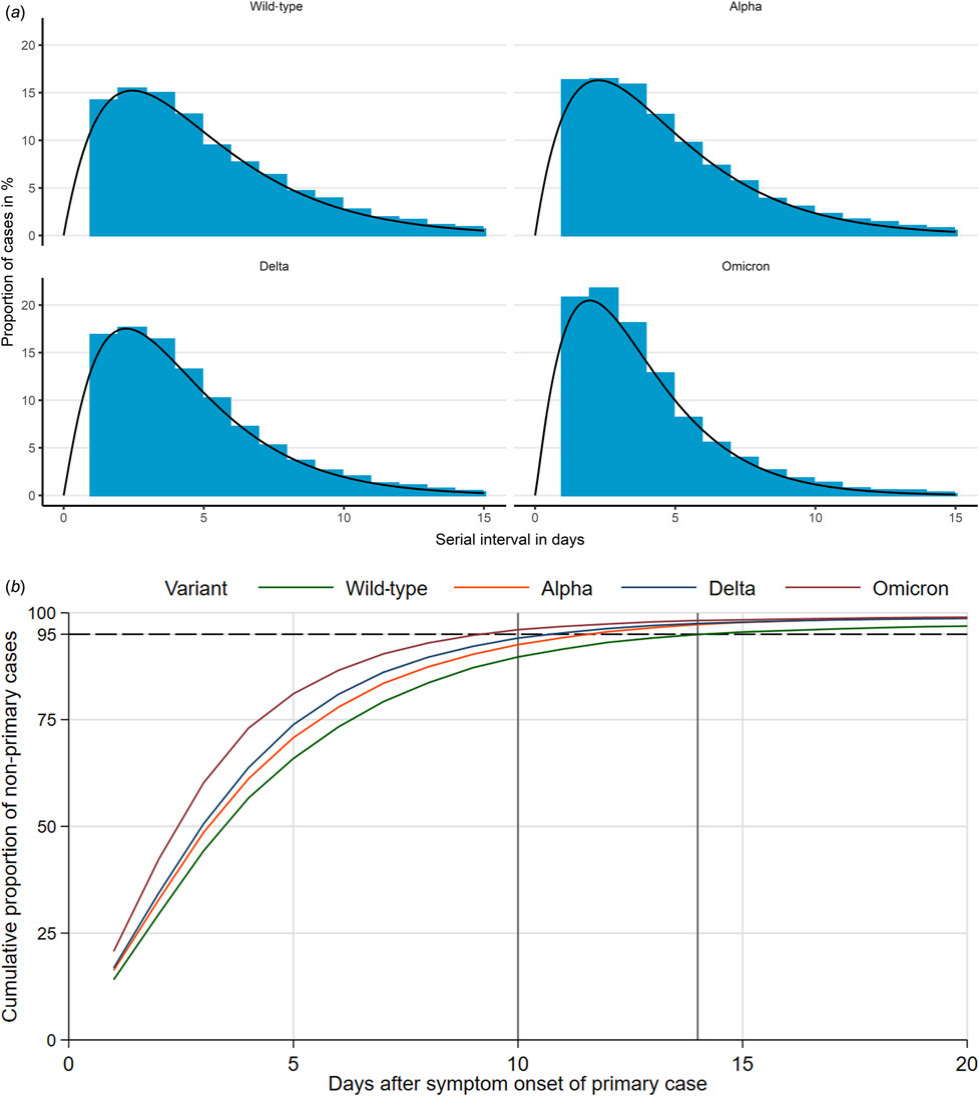
(a) Distribution of serial interval from first to secondary cases in households by variant and fitted *γ* distribution. (b) Cumulative proportion of non-primary cases in households, in days after symptom onset of primary case and by variant; vertical lines at 10 and 14 days, dashed horizontal line at 95%. The estimated parameters for the Gamma distribution were for Wild-type, Alpha, Delta and Omicron respectively shape (2.02, 2.00, 2.11, 2.14) and rate (0.42, 0.44, 0.50, 0.59).

The mean serial interval dropped from 4.80 days (95% confidence interval (CI) 4.74–4.85) during wild-type circulation to 4.50 (95% CI 4.46–4.54) during Alpha, 4.19 (95% CI 4.16–4.22) during Delta and 3.61 (95% CI 3.56–3.66) during Omicron circulation (Table 1). All differences are statistically significant. Including day 0 secondary cases would lower these mean values by 0.6–0.75 days, but leave the differences similar. In Omicron households 95% of secondary cases occurred until day 10 after symptom onset of the primary case, compared to day 11, 12 and 14 for Delta, Alpha and wild-type, respectively (Fig. 1b). The cumulative probability function shows only a slight dependence on the total number of cases by household. We observe a U-shape of the mean serial interval as a function of the first cases age for the wild-type and Alpha (Fig. 2).

**Table 1.** Mean time from symptom onset of primary case in households and symptom onset of secondary case, by variant; Germany, 2020–2022

	Time in days	Lower 95% CI	Upper 95% CI
Wild-type	4.80	4.74	4.85
Alpha	4.50	4.46	4.54
Delta	4.19	4.16	4.22
Omicron	3.61	3.56	3.66

**Fig. 2.**
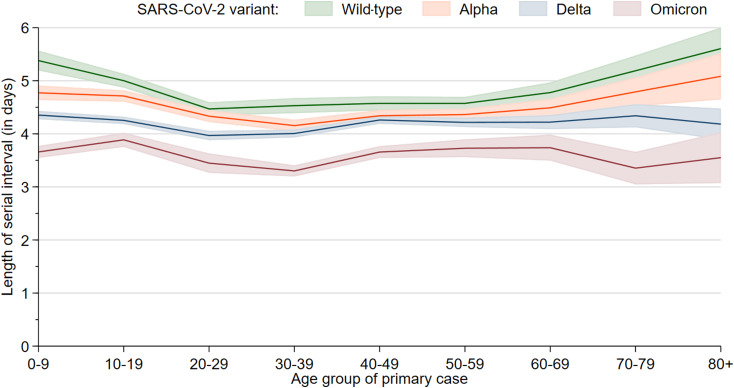
Mean length of serial interval from first to secondary cases in households as a function of the first cases age group, by variant, with 95% confidence bands.

In a subanalysis we also investigated the serial interval for the lineages BA.1 and BA.2 of Omicron and found mean values of 3.88 days (95% CI 3.79–3.97) and 3.39 days (95% CI 3.30–3.49). Here, we used all cases in household outbreaks of CW 2–5 for BA.1 and CW 11–16 in which BA.2 was dominating in Germany.

Thus, serial interval of household cases seems to have shortened even more for the Omicron variant compared to any of the variants before. This trend seems to continue even for the lineages of Omicron, BA.1 and BA.2. Results of our analysis are consistent with those from Abbott *et al*. who estimated a mean generation time for Omicron as 2.5–4 days (90% credible interval) using surveillance case counts [4] and the UK who reported a serial interval from non-household contact tracing data of 3.72 days for the BA.2 lineage of Omicron and 3.27 days for the BA.1 lineage [5]. Backer also analysed household data and reported a 4.1 days serial interval for Delta and a 3.5 days serial interval for Omicron [6]. Compared to Backer our estimates are slightly longer perhaps because we excluded systematically potential secondary cases when they had symptom onset on the same day as the assumed primary case. Moreover, due to our definition of the first case negative serial interval was not possible.

The U-shape of the mean serial interval as a function of the first cases age for the wild-type and Alpha might reflect the shorter and less strong viral shedding of children and the less intense contact of older household members inside the household. This pattern seems to disappear for Delta and Omicron and we see a more constant shape over the age groups. A possible explanation might be a more effective transmission that also results in higher attack rates inside the households, especially for Omicron.

We wish to mention two limitations of our analysis. First, we included only data from households where symptom onset was known for all household cluster cases. We assumed that the case with the first onset of symptoms was the primary case. However, it is possible that, for example, asymptomatic persons, in particular children, have occasionally been the primary case infecting other household contacts pulling the distribution inadvertently towards the primary case. Nevertheless, generally persons with asymptomatic SARS-CoV-2 infection tend to be substantially less infectious than symptomatic persons [7–9] implying that – by and large – our assumption should hold. In addition, children were increasingly screened since approximately the summer of 2021 so that asymptomatic children were probably for the most part detected. Second, as stated we excluded cases with a symptom onset identical to that of the primary case. Should we have opted to include these persons it would have shortened the serial interval even more.

In summary, after the serial interval of the previous variants (Alpha, Delta) has already decreased we believe that there are clear indications that the serial interval of Omicron has shortened even further.

## Data Availability

The research presented in this paper is based on data that were routinely collected by LPHA and reported the Robert Koch-Institute in accordance to §4 of the German Protection against Infection Act (IfSG, BGBl. I S. 1045). Due to privacy regulations, the data are securely stored at Robert Koch-Institute and cannot be made available to the public by law.
